# Supplementary material for: Freshwater budget in the Persian (Arabian) Gulf and exchanges at the Strait of Hormuz
Source: PLoS One. 2020 May 29;15(5):e0233090. doi: 10.1371/journal.pone.0233090 (PMC7259735; doi:10.1371/journal.pone.0233090)
Supplement: Attachment — Submitted filename: Response to Reviewers.pdf [file pone.0233090.s001.pdf]

AUS | الجامعة الأمريكية في الشارقة
American University of Sharjah

Sharjah, 26 April 2020

Dr. João Miguel Dias, Ph.D.

Academic Editor

PLOS ONE

Re: PONE-D-20-06928

Dear Sir

This is a Rebuttal Letter to the reviewers of the manuscript entitled "Freshwater budget in the Persian (Arabian) Gulf and exchanges at the Strait of Hormuz" submitted for publication in PLOS ONE.

We greatly appreciate all the suggestions and inform that we have done a complete revision of the paper, making all the appropriate changes and additions proposed by the reviewers, as itemized below.

Yours truly,

Dr. Edmo Campos

REVIEWER #1

1) We inserted a definition for Sv (Sverdrup) in the abstract;
2) Following his suggestions, we reduced to a minimum the number of acronyms throughout the text, leaving only those deemed necessary and with the appropriate and clear definition;
3) As recommended, we inserted the definition for SST (sea surface temperature), at its first appearance in the text;

4) We modified the text, adding references and a few words about the potential importance of the Gulf waters in the Indian Ocean.

5) Section 5 was slightly reduced, following the reviewer's recommendation.

REVIEWER #2

We sincerely acknowledge the excellent review, including detailed editing suggestions and comments throughout the manuscript, which we gladly accepted and contributed to the paper's readability and quality. Below we itemize these changes, following the list in the reviewer's comments. As for his major concerns, our response is the following.

1) **The freshwater term**. We apologize for not having made it clearer. This terminology is used because in the paper it is exactly hyposaline or water with zero salinity. The freshwater we refer to is the amount of water with no salt content that would correspond to the amount of salt transported in the opposite direction. Or, as explained by Drijfhout et al (2011): *the equivalent freshwater budget obtained by dividing the equation for salt transport across a vertical section by a reference salinity* (the quantity M_t in Eqs. 4 and 5, as explained on lines 265-266 of the original manuscript). To help the reader to better understand it, we included an explanation in the new version of the abstract.

2) We changed slightly the *Abstrac*t, to include some words making a better promotion of the most relevant findings.

3) Thanks for pointing out this detail. We revised the complete manuscript and include the names when referring to specific authors.

Response to the reviewers #2 general comments:

Some of the points, listed below, were just editorial suggestions, typos or other minor comments. They were all accepted, with the appropriate change made in the new version.

Lines 4, 18, 22, 23, 24, 31, 33, 35, 42, 45, 46-48, 56-59, 68, 71, 72, 74-94, 107, 107-115, 119, 133-134, 138, 145-6, 151, 180, 184, 218, 226, 233, 275, 278, 364, 365, 483, 485, 492.

These are comments that required more elaborate responses.

Line 10: "what" was replaced with "which" and the verb was put in the plural ("make") to agree with the subject: the warmer and more buoyant characteristics of the waters.

Line 209: The **freshwater issue**, explained in Item (1) above

Fig. 3: We improved the figure and changed the caption, to include the layer mean depth.

Fig. 4: We included the mean depth in the caption and in the main text.

Fig 5: The blue lines were explained in the caption.

Fig 6: The red line explained in the caption.

Line 342: The reviewer is right. The model considers the mean depth in each 7km cell. However, in the vicinity of the point considered, the bathymetric gradient is not high. The major concern is with the bathymetry of a larger area, including the northernmost region of the Arabian Peninsula. The ETOPO5 bathymetry does not resolve it properly (we are currently running a version of the model, with a much higher horizontal resolution (1/48 of a degree) and improved bathymetry (GEBCO 15arcsec bathymetry).

Lines 343-4: We apologize for not being clearer. We changed the text to better explain what we meant. Our model products are 6-day averages and the data collected by Johns et al (2003) have higher resolution. We do not have access to that data. In the future, we plan to analyze a higher frequency output of the higher-resolution model's implementation.

Lines 393-398: We gladly accepted the suggestion and included some text with respect to the desalination activity. As a matter of fact, this is part of our ongoing investigation, in which we address the question.
